# Microsatellite Diversity and Phylogenetic Relationships among East Eurasian *Bos taurus* Breeds with an Emphasis on Rare and Ancient Local Cattle

**DOI:** 10.3390/ani10091493

**Published:** 2020-08-24

**Authors:** Gulnara Svishcheva, Olga Babayan, Bulat Lkhasaranov, Ariuntuul Tsendsuren, Abdugani Abdurasulov, Yurii Stolpovsky

**Affiliations:** 1 Vavilov Institute of General Genetics, Russian Academy of Sciences, 119333 Moscow, Russia; stolpovsky@mail.ru; 2 Institute of Cytology and Genetics, Siberian Branch of the Russian Academy of Sciences, 630090 Novosibirsk, Russia; 3 Gordiz Ltd., Skolkovo Innovation Centre, 121205 Moscow, Russia; babayan@gordiz.ru; 4 Shuluuta Ltd., 671450 Kizhinga, Russia; lbulad@mail.ru; 5 Institute of General and Experimental Biology, The Mongolian Academy of Sciences, Ulaanbaatar 210351, Mongolia; aakii55@yahoo.com; 6 Department of Agriculture, Faculty of Natural Sciences and Geography, Osh State University, 723500 Osh, Kyrgyzstan; abdurasul65@mail.ru

**Keywords:** microsatellites, diversity, cattle, origin, breeding

## Abstract

**Simple Summary:**

Using microsatellite data, we analyzed various cattle breeds of European and Asian origins from different geo-climatic zones to study their genetic diversity, genetic distances, population structure, and other characteristics. The main focus was on the analysis of ancient and rare native breeds, which either have been unexplored or have received very little attention: the Altai, Ukrainian Grey, Tagil, and Buryat cattle breeds. Our findings provide important information on the population origin and diversity of the breeds, which can be useful for breeding and conservation purposes.

**Abstract:**

We report the genetic analysis of 18 population samples of animals, which were taken from cattle (*Bos taurus*) breeds of European and Asian origins. The main strength of our study is the use of rare and ancient native cattle breeds: the Altai, Ukrainian Grey, Tagil, and Buryat ones. The cattle samples studied have different production purposes, belong to various eco-geographic regions, and consequently have distinct farming conditions. In order to clarify the genetic diversity, phylogenetic relationships and historical origin of the studied breeds, we carried out an analysis of the genetic variation of 14 high-variability microsatellite loci at 1168 genotyped animals. High levels of heterozygosity and allelic richness were identified in four of the ancient local breeds, namely the Kalmyk, Tagil, Kyrgyz native, and Buryat breeds. The greatest phylogenetic distances from a common ancestor were observed for the Yakut and Ukrainian Grey breeds, while the Tagil breed showed the smallest difference. By using clustering approaches, we found that the Altai cattle is genetically close to the Kyrgyz one. Moreover, both the Altai and Kyrgyz breeds exposed genetic divergences from other representatives of the Turano-Mongolian type and genetic relationships with the Brown Swiss and Kostroma breeds. This phenomenon can be explained by the extensive use of the Brown Swiss and Kostroma breeds in the breeding and improvement processes for the Kyrgyz breeds, which have been involved in the process of keeping the Altai cattle. Our results can be valuable for conservation and management purposes.

## 1. Introduction

In recent years, significant progress on the individual identification and certification of breeds has been made in most countries for major livestock species. This was primarily achieved by the use of microsatellites, which have become internationally preferred molecular markers to trace studbook information at levels from a species to an individual. Microsatellites distributed not very densely but evenly throughout the eukaryotic genome [1] are hypervariable due to variation in the number of repeat units [2,3,4,5]. Because of a high level of polymorphism and a relatively uniform coverage across the genomes, microsatellite markers have proven to be an extremely valuable molecular tool for a wide range of genetic studies in humans, model organisms, wild vertebrate populations, and agriculturally-important animals [6,7,8,9,10], including breeds of cows [5], horses [11], goats [12], and pigs [13].

The International Society for Animal Genetics (ISAG) and the Food and Agriculture Organization (FAO) of the United Nations have proposed to use microsatellites in population genetics studies of livestock breeds, in particular, to analyze their genetic diversity, genetic distances, population structure, breed purity, breed origin, effective population size, and other characteristics [14]. Different levels of multilocus heterozygosity and allelic diversity revealed in the early studies related to the microsatellite polymorphism analysis in the cattle have provided impetus for a whole series of studies in this field (see, for example, [5,8,15,16,17,18,19,20,21,22,23,24]). Most studies have been performed on local and transboundary cattle breeds with a wide breeding range, helping address both applied (characterization and monitoring of animal genetic resources) and fundamental (genome mapping, phylogeny, molecular genetics classification, gene geography, genetic variability analysis, etc.) problems of cattle breeding. For example, a meat traceability system has been developed for indigenous breeds of Chinese cattle to ensure meat safety and to solve adulteration issues [23]. For distinct ecotypes of Nguni cattle adapted to very different environmental regions of South Africa, a close genetic relationship has been reported [20]. The local Macabea cattle has been found to belong to the American Creoles group and has a south Spanish origin [5]. Finally, genetic uniqueness and allelic diversity have been found in indigenous Korean cattle breeds compared to international and regional cross-border breeds [22].

In recent years, significant advances have been made in whole-genome sequencing in domestic animals [25] and primarily in cattle [26]. In particular, the genetic diversity of a large number of European cattle breeds has been studied by whole-genome genotyping arrays containing ~ 50 × 10^3^ –150 × 10^3^ single nucleotide polymorphisms (SNPs) [27]. Although SNPs are located densely enough throughout the genome and have a low genotyping error rate [28,29], they do not fully explain the observed genetic diversity, because new SNP variants (~2.5 × 10^−8^ SNPs versus 10^−2^–10^−5^ microsatellites per generation) arise more rarely than new microsatellite variations [30]. Therefore, microsatellites are suitable markers to provide information complementary to that gained using SNPs [31]. The strongest argument for the above statement is the fact that they are presented in the genome as neutral elements that do not have a distinct biological function and evolve rapidly, which allows them to quickly accumulate mutations [32,33,34].

**Figure 1:**
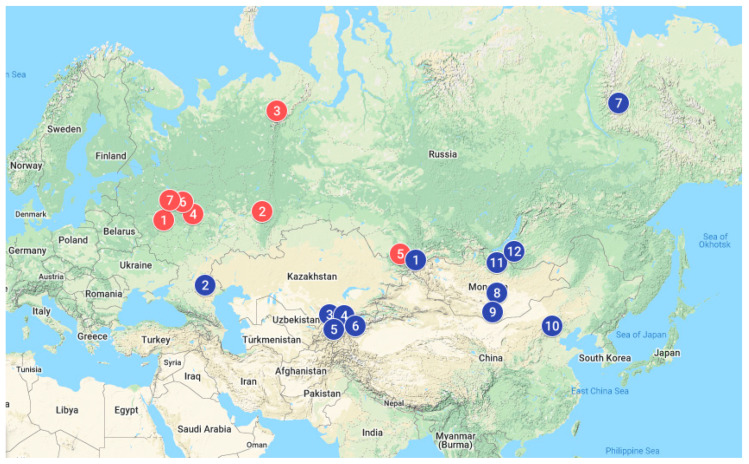
Geographic locations of cattle breeds. A total of 18 cattle breeds were sampled in this study, including eight European breeds indicated by red dots ((1). Holstein, (2). Tagil, (3). Kholmogory, (4). Red Gorbatov, (5). Ukrainian Grey, (6). Yaroslavl, (7). Brown Swiss and Kostroma) and ten Asian breeds indicated by blue dots ((1). Altai, (2). Kalmyk, (3). Aulie-Ata, (4). Kyrgyz Beef-type, (5). Alatau, (6). Kyrgyz native, (7). Yakut, (8). Khogorogo, (9). Gobi breeds, and (10–12) Buryat from China, Mongolia and Russia, respectively). (The map was downloaded from https://www.google.com/maps).

The purpose of this study was to use microsatellite variation in the European and Asian cattle (*Bos taurus*) breeds for assessing their genetic diversity and addressing whether their gene pool reflects their historical origin. We analyzed cattle samples raised in different eco-geographic regions (Figure 1). These samples come from distinct environmental and farming settings and differ in terms of productivity traits, economic importance, population status (endangered, vulnerable, or not at risk), and phylogenesis.

The analysis was focused on four most ancient and rare native breeds: Ukrainian Grey (imported in 1982 to Russian Altai from the Ukraine) [35], Tagil (Russia) [27], Altai (Russia), which is officially classified as ‘extinct’, and Buryat (a mixed sample from Buryatia, Mongolia, and Inner Mongolia) [27,36]. Note that the Altai and Buryat breeds were not mentioned in the cattle breed census carried out in Russia in the 20th century and have been poorly investigated at the molecular genetic level. To be sure, the Altai and Buryat cattle are of interest in terms of the phylogeny of cattle breeds in general.

The Buryat native cattle is an independent breed with a long history of natural selection and selective breeding in the extreme continental climatic conditions of Buryatia. This breed is characterized by health promoting properties, high disease resistance (it has never been diagnosed for tuberculosis or leukemia), and high-quality meat. The Buryat cattle breed was considered extinct before a limited number of animals (~200) were recently found in remote areas of Mongolia and Inner Mongolia. Some of these animals were taken to Buryatia (Russia) for breeding [27]. Now an effort is under way in Buryatia to restore and conserve the genetic resources of Buryat native cattle. Since a preliminary analysis of samples of Buryat cattle from all these three geographical regions did not reveal genetic differences between them (data not shown), we included in the analysis a combined sample of Buryat cattle. As for the native Altai cattle, there is virtually no information about it in the literature. It is only known that it was undersized pasture cattle (without stall housing) isolated geographically by the Altai Mountains. Animals lived in the pasture all year round. The Altai cattle was as well adapted to the harsh conditions of a sharply continental climate as the Buryat breed. Recently, an isolate with a small number of local livestock was found in a hard-to-reach area of the Ulagan district of the Altai Mountains. The Altai cattle is kept by the Telengits, an ethnic group which shares a common origin with the Kyrgyz. This cattle is small, and its coat has various combinations of four basic colors (red, brown, white, and black). Additionally, the Altai native cattle is similar to dual-purpose (dairy/beef) breeds. In this study, the Altai cattle is represented by animals from this geographic isolate. In our study, we included Yakut native cattle breed in the Sakha Republic (Russia) (see, for details, [8,37,38,39]). The Yakut breed is the last remaining native Turano-Mongolian-type cattle in Siberia. This breed is characterized by high endurance, because it has better mechanisms of adaptation to extreme climatic conditions. The animals are able to consume and digest large amounts of roughage and are resistant to many diseases inherent in other cattle breeds [40]. Additionally, we analyzed Red Gorbatov, Kholmogory, and Kostroma, which are among the breeds considered by FAO as being at risk of extinction. More details about cattle breeding and breed conservation can be found in the reference works [41,42].

## 2. Materials and Methods

### 2.1. Sample Information and Microsatellite Data

In this study, the genotypes of 14 highly stable and polymorphic microsatellites (BM1824, BM2113, CSRM60, CSSM66, ETH3, ETH10, ETH225, ILSTS006, INRA023, SPS115, TGLA53, TGLA122, TGLA126, and TGLA227) were determined. The description of all the markers used and their distribution along 30 cattle chromosomes was reported by [43] (Table A1). All these loci, with the exception for CSSM66, ILSTS006, and CSRM60, are included in the panel recommended by ISAG for genetic diversity studies in cattle.

The genotypes of 1168 animals representing 18 cattle breeds from three countries (Russia, Kyrgyzstan, and Mongolia) were analyzed. The samples were collected from ten breeds of Asian origin (Khogorogo and Gobi (Mongolia), Buryat (a mixed sample from Russia, Mongolia, and Inner Mongolia), Kalmyk, Yakut, and Altai (Russia)) and eight breeds of European origin (Kostroma, Kholmogory, Red Gorbatov, Yaroslavl, Tagil, Brown Swiss, Holstein, and Ukrainian Grey (Russia)) (Table 1).

Noteworthy, the majority of the studied cattle breeds was created by crossing native cattle with European breeds. For instance, the Kostroma cattle was developed by crossbreeding the improved native cattle from the Kostroma region (Russia) with predominantly the Brown Swiss and Allgau breeds. The Tagil breed was developed from Ural native primitive cattle through multistep hybridization with breeds of Dutch origin, namely Dutch, Kholmogory, and Red Gorbatov. The Aulie-Ata breed was formed in Kyrgyzstan on the basis of the Kyrgyz native cattle crossed with Dutch Black Pied. The Alatau (sometimes spelt as ‘Ala-Tau’) breed was created on Kyrgyzstan’s farms by crossing Kyrgyz native cattle with Swiss Brown and Kostroma and selective breeding. By crossing Alatau cows with bulls of the Aberdeen-Angus breed, the Kyrgyz beef-type cattle was developed.

### 2.2. DNA Extraction and Fragment Analysis

Standard methods for DNA extraction from blood samples, genotyping, and allele calling were used. DNA was extracted from blood samples using the Diatom™ DNA Prep 200 reagent kit (Isogene Lab Ltd., Moscow, Russia) according to the manufacturer’s recommendations.

The microsatellite data were presented as a two-dimensional matrix, whose elements were the genotypes of microsatellite loci. For each animal and for each locus, the genotype was scored in terms of the allele length, i.e., the number of sequenced bases within a read separating non-repetitive flanking boundaries aligned to the reference, no matter what intervening alignment gaps. The percentage of missing genotypes was about 0.1%. Microsatellite analysis was carried out by the biotechnological company Gordiz certified as meeting ISO 9001: 2015. Multiplex PCR analysis of microsatellite loci containing short tandem repeats was performed using the COrDIS Cattle set (Gordiz, Moscow, Russia) according to the manufacturer’s instructions. The PCR amplification was performed using the Applied Biosystems Veriti thermal cycler (Thermo Fisher Scientific, Waltham, MA, USA).

The primers for PCR were selected so as to enable the simultaneous amplification of all 14 loci in a single tube. Each amplified PCR product was less than 320 base pairs in size. PCR products were analyzed by capillary electrophoresis with laser-induced fluorescent detection. The COrDIS Cattle kit uses five fluorescent dyes characterized by different emission wavelengths to allow simultaneous detection in different fluorescence channels. Primers were labeled with four fluorescent dyes (Blue, Green, Yellow, and Red). The S450 size standard was labeled with the fifth fluorescent dye and detected in a separate channel (Orange) simultaneously with the PCR products. To obtain the complete STR-profile of any sample, it is sufficient to have 0.2 ng of nondegraded DNA.

### 2.3. Statistical Analysis

Calculations were performed in the R environment, version 3.6.1 (R Core Team, 2019, Vienna, Austria). The adegenet R package [44] was used to calculate the basic population genetics statistics for each marker and sample, including the observed number of alleles, number of alleles per locus, and number of alleles per sample. The distribution of alleles across loci and samples was calculated using the diveRsity R package [45]. Testing for Hardy–Weinberg equilibrium for each combination of sample and locus was performed using the PopGenReport R package [46,47]. Using the same package, genetic variability indices such as allelic richness, allele frequencies, and the number of private alleles were calculated. In particular, allelic richness was estimated by the rarefaction-based method of Mousadik and Petit [48], implemented in the allelic.rich() function, since the sizes of the studied samples differ from each other. Using the formula proposed by [49], polymorphism information content (PIC) was estimated for each locus and sample on the basis of the number and frequency of alleles at the locus in the polysat R package [50]. For each sample, the observed and expected heterozygosity, fixation index, and *p*-value for the Hardy–Weinberg equilibrium test were calculated using the diveRsity R package. Nei’s Pairwise Fst values were calculated using the pairwise.fst() function from the hierfstat R package [51]. The parameters of allelic diversity, described in [52,53], were computed by the Metapop2 software [54], and allelic distances between samples were calculated by the allele.dist() function from the PopGeneReport R package. Breed differentiation was analyzed using the Bayesian clustering approach implemented in Structure v. 2.3.4 software [55]. This program generates clusters of individuals based on their multilocus genotype data. The optimal number of clusters was determined using the method proposed by [56] and the Structure Harvester program [57]. The phylogenetic tree based on Nei’s genetic distances [58] was inferred using the poppr R package [59] with bootstrap support from 5000 replicates.

## 3. Results

### 3.1. Genetic Variability

For the 14 microsatellites analyzed, a total of 192 alleles were detected, with 9 (TGLA126 and ETH10) to 25 (TGLA122) per locus. The mean number of alleles per locus across all samples was 13.7. The average percentage of the total number of alleles observed in the locus varied from 35.9% (Yakut breed) to 81.5% (Buryat breed) (Table 2). We observed a significant (*p*-value < 2.0 × 10^−4^ with the Bonferroni correction) departure from the Hardy–Weinberg equilibrium (Table A2) for only two samples, namely, the Buryat (ILSTS006) and Brown Swiss (BM2113, TGLA227, CSSM66, and TGLA53).

We calculated allele frequencies (AF) and the PIC values as a measure of the amount of information that can be recovered from a genetic marker. High AF values (AF > 0.7) were obtained only for four samples, namely, Red Gorbatov (SPS115.254), Yaroslavl (SPS115.254), Kostroma (TGLA126.119), and Yakut (INRA023.204, SPS115.261, and BM1824.186). For each locus and each sample, the PIC values were estimated on the basis of the number and frequency of alleles at the locus (Table A3). The mean PIC value appeared to be rather high, 0.77 ± 0.02. The highest polymorphism levels were obtained for TGLA53 (PIC = 0.903), TGLA227 (PIC = 0.870), CSSM66 (PIC = 0.837), TGLA122 (PIC = 0.822), and BM2113 (PIC = 0.817). To be sure, the genetic individuality of a breed may be defined by private (potentially breed-specific) alleles. Out of 192 alleles in 1168 animals genotyped, 16 alleles were private: one in Ukrainian Grey (AF = 0.011, n = 44), and the others in five Asian native breeds, including Buryat with nine private alleles (mean AF = 0.010 ± 0.003, n = 286) (Table 3). The largest number of private alleles per locus was detected for TGLA227 (3). The largest numbers of private alleles adjusted for sample size were in the Kalmyk and Kyrgyz native cattle (18% and 19.9%, respectively of their total number). The presence of private microsatellite alleles with frequencies above 0.01 in the native cattle breeds suggests that each of these breeds most likely has a unique gene pool. No private alleles were detected in the international or regional transboundary breeds (Holstein, Yaroslavl, Red Gorbatov, Brown Swiss, and Kholmogory).

Genetic variability in each animal sample was studied in terms of the number of alleles (A), allelic richness (Ar), observed (Ho) and expected (He) heterozygosity, fixation index (Fis), and *p*-value for the chi-square test of the Hardy–Weinberg equilibrium (HWE) (Table 2). Ar varied from 4.29 (Yakut breed) to 7.52 (Alatau breed) with a mean of 6.15 ± 0.23. Because allelic richness is more sensitive to the loss of rare alleles and differences in sample size than expected heterozygosity, it is more useful in identifying genetic bottlenecks [60,61]. We explored the relationship between Ar and He and revealed a significant correlation (0.918) with the determination coefficient R^2^ = 0.842 (*p*-value = 8.275 × 10^−8^). The observed and expected heterozygosity values were compared using the Bartlett test. The results obtained showed that there was a difference between the mean Ho and He values, and the pooled sample deviates from the Hardy–Weinberg equilibrium (*p*-value = 0.8895). High Ho values (0.78) were found for Red Gorbatov and Kalmyk, and the lowest (0.61), for Yakut. We calculated the fixation index, Fis, for each sample as Fis = (He-Ho)/He (Table 3). High Fis values point to a decreased heterozygosity of the microsatellites due to inbreeding. A very slight deficiency of heterozygotes was observed in two samples: Aulie-Ata (Fis = 0.0128) and Buryat (Fis = 0.013). The Fis values were equal to zero for Kholmogory, Khogorogo, Gobi, Kalmyk, and Alatau. This implies that mating within the breeding farms is absolutely random and non-assortative, and no inbreeding occurs. As far as the other breeds are concerned, we observed an excess of microsatellite heterozygotes. The mean Fis value in the pooled sample is −0.026 ± 0.009.

### 3.2. Pairwise Fst and Ast Values

Pairwise Fst values were calculated and used to estimate the level of genetic differentiation between the populations among all the loci (Table A4). For genetic differentiation, several levels of significance were examined (*p*-values = 0.05; 0.01 and 0.005). We found that most of the samples studied are significantly differentiable relative to each other in terms of Fst. However, for some pairs of samples, genetic differences were not found. For example, the samples of Kyrgyz native and Alatau cattle were most closely genetically related to each other (Fst = 0.0049, *p*-value = 0.7552). This fact is consistent with historical data relating these Turano-Mongolian-type breeds. Moreover, the Buryat breed showed non-significant or weak genetic differentiation with one European sample, the Tagil breed (Fst = 0.0087, *p*-value = 0.1209), and with all the samples of Asian origin (Fst values range from 0.0073 to 0.0165, *p*-values > 0.005), with the exception of the Yakut breed (Fst = 0.0231, *p*-values = 0.001). The Yakut breed was the most distant from all the other breeds and most so from Ukrainian Grey and Kostroma, with the respective pairwise Fst values being equal to 0.1459 and 0.1527 (all *p*-values = 0.001).

As an alternative to pairwise Fst, we calculated the Ast values as indices of allele differentiation between samples (Table A5). It was detected that the lowest Ast values were among the Kyrgyz native, Kyrgyz beef-type, and Holstein breeds (Ast < 0.1799), and the highest Ast was between the Yaroslavl and Gobi breeds (Ast = 0.3447). Besides, the last two showed high allelic differentiation with all other studied breeds (Ast > 0.2747 for Gobi, and Ast > 0.2840 for Yaroslavl), with the allelic diversity averaged over all breeds of 0.2358. We calculated the average allelic diversity within samples (As = 5.5994) and between samples (Da = 1.6949), with the total allelic diversity (At = As + Da) of 7.2944.

In addition, for each pair of population samples, we estimated allelic distance (denoted here as Adis) as the number of alleles present in one sample and absent in the other [62,63] (Table A6). The greatest allelic distance was revealed between Buryat and Yakut native cattle (Adis = 48.5, *p*-value = 0.001). Ukrainian Grey cattle showed allelic dissimilarities with all the studied breeds (Adis > 26.5, *p*-value < 0.041), except the Brown Swiss one. It is noteworthy that Altai cattle significantly differs from all the Turano-Mongolian type breeds (Adis > 26.5, *p*-value < 0.024), except the Gobi one (Adis = 20, *p*-value = 0.384). In general, we did not find significant strong allelic dissimilarities among four the Kyrgyz breeds coming from Kyrgyzstan’s indigenous cattle (Adis < 23.5, *p* > 0.059), as well as in the group of European cattle (Adis < 31, *p* > 0.01), with the exception of the Ukrainian Grey breed.

### 3.3. Wright’s F Statistics for Each Locus

Among 14 microsatellites, the values of the overall inbreeding coefficient, Fit, ranged between 0.0333 and 0.0935 (Table 4) with a mean of 0.0681, suggesting that these cattle breeds have low inbreeding rates. The values of the inbreeding coefficient, Fis, varied from −0.0349 to 0.0059 with a mean of −0.0085. The negative values of Fis indicate that heterozygosity either is ‘excessive’ or does not conform with the Hardy–Weinberg proportions [64]. The estimates of the fixation index, Fst, ranged from 0.0608 to 0.0955 with a mean of 0.076, suggesting a high level of genetic differentiation among the cattle samples and determining the contribution of each locus to the genetic differentiation of the breeds. The greatest contribution was made by the ILSTS006, SPS115, BM1824, and TGLA122 microsatellites with Fst values greater than 0.08.

### 3.4. Bayesian Clustering Analysis

**Figure 2:**
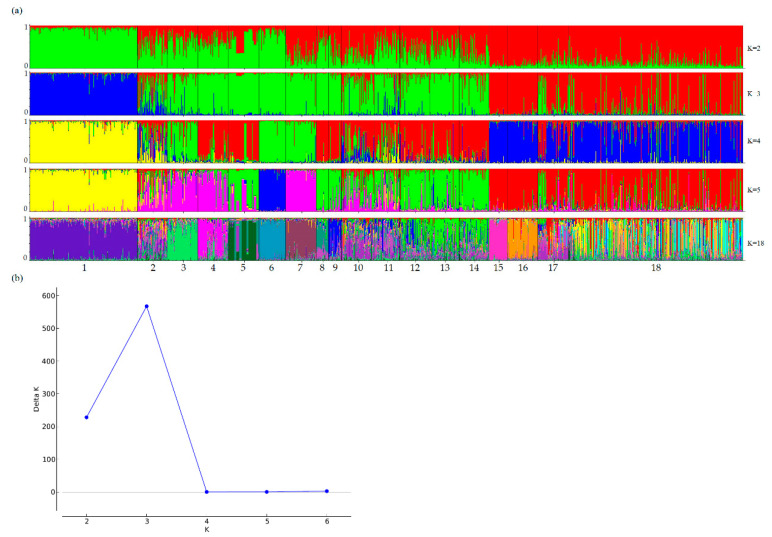
Results of Structure analysis based on microsatellite data: (**a**) proportion of membership of each animal to K assumed clusters (K  =  2–5, 18). Breed numbering: Holst (1), Tagil (2), Kholm (3), RedGor (4), BrSwis (5), Grey(6), Yaros (7), Kostr (8), Altai (9), Kalm (10), AulieAt (11), Alatau (12), KyrgBT (13), KyrgNat (14), Yakut (15), Khogor (16), Gobi (17), and Buryat (18); (**b**) values of the statistic ΔK calculated by the method proposed by Evanno et al. for K = 1–7.

A Bayesian clustering approach based on the Markov chain Monte Carlo (MCMC) simulations was employed to assess the population structure. This method uses multilocus genotypes as input data to infer the fraction of population/individual genetic ancestry that belongs to a cluster, for a given number of clusters (K). We performed 60 runs for each K from 1 to 7, while further increasing K (K > 7) did not lead to any significant progress. We chose an admixture model with correlated allele frequencies. This model assumes that an animal may have a mixed origin, i.e., a fraction of its genome may have been inherited from a particular cluster. This model allows us to identify hybrid animals and even animals that actually belong to other breeds. This model assumes that the samples within each cluster are in the Hardy–Weinberg equilibrium for each of the markers tested. To choose the optimal number of clusters, a burn-in period of 100,000 generations and 1,000,000 iterations of MCMC simulations were used in all the above-mentioned runs. Using the method proposed by Evanno et al., which is based on the ad hoc statistic ΔK, we determined the optimal number of clusters as K = 3 (green, blue, and red clusters in Figure 2).

The first cluster consists only of the Holstein cows (blue bars), the second cluster includes four Turano-Mongolian-type native breeds (Yakut, Buryat, Gobi, and Khogorogo) (red bars), while the third cluster (green bars) is mixed—it comprises breeds of both European and Asian origin. At K = 18, when the maximum proportion of a sample’s membership in any of these clusters (Pm) is above 80%, we acknowledge ‘pure ancestry’. Pure ancestry was thus detected at four samples: Yakut (89%), Ukrainian Grey (87%), Holstein (83%) and Yaroslavl (81%). Other samples were found to have a mixed ancestry. The Kyrgyz beef-type, Gobi, Buryat, and Tagil samples had the lowest Pm values (<20%).

### 3.5. Phylogenetic Analysis

**Figure 3:**
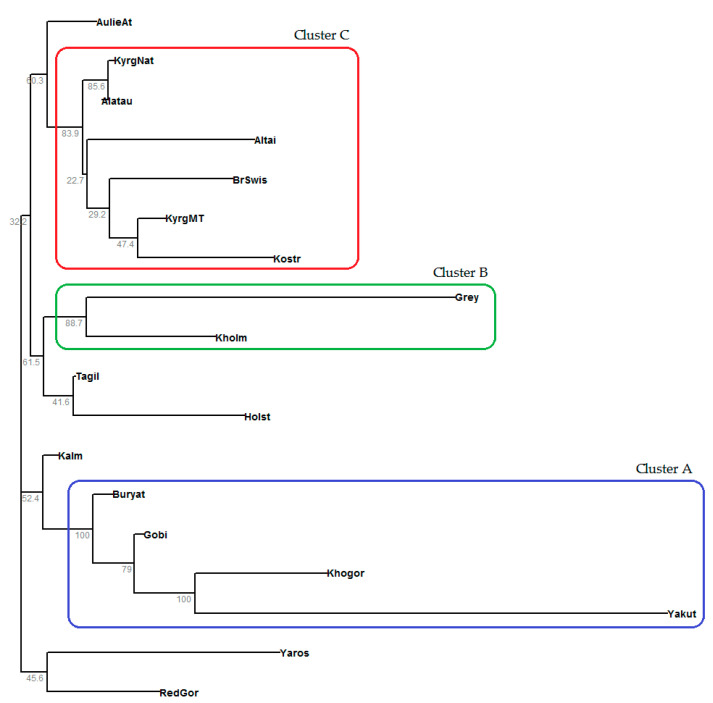
Phylogenetic tree constructed by the neighbor-joining algorithm using Nei’s genetic distances. Numbers at the branch nodes indicate the confidence values for each branch obtained using the bootstrap procedure. The red, green, and blue rectangles show three clusters of the livestock breeds with BS > 80%.

Using Nei’s genetic distances [58], we reconstructed a rooted phylogenetic tree by the neighbor-joining algorithm, which allows for unequal rates of evolution (Figure 3).

We found three breed clusters with high bootstrap support (BS) and several separate breeds that were not included in any cluster. The cluster A is formed only by Turano-Mongolian-type breeds [65], namely Yakut, Khogorogo, Gobi, and Buryat (BS = 100%). The Kalmyk cattle (which is a Turano-Mongolian-type, too) adjoins the cluster A, but with 52.4% BS. The entries in this cluster confirm the common historical roots of these five breeds. The cluster B includes two breeds of European origin (BS = 88.7%): Ukrainian Grey and Kholmogory, which are of the same type according to the craniological classification [35]. It is quite expected that the Tagil cattle is grouped with the Holstein one and together they adjoin the cluster B, but with 61.5% BS, since European breeds, mainly Holstein and Kholmogory bulls, were involved in crossings with Tagil cows to improve their milk productivity. The cluster C consists of two brown breeds of European origin (Brown Swiss and Kostroma) and four breeds of Asian origin (Altai cattle, Kyrgyz native, Kyrgyz beef-type, and Alatau breeds), with 83.9% BS. Furthermore, the Aulie-Ata breed is a member of the cluster C, but with 60.3% BS. Clustering the Kostroma, Brown Swiss, and Alatau breeds is consistent with the well-known history of the formation of these cattle breeds. The presence of all the Kyrgyz breeds in one cluster is not surprising, for they all come from Kyrgyzstan’s indigenous cattle. Note that the Kyrgyz native and Alatau breeds are the closest within this cluster (BS = 85.6%). Thus, a heterogeneous composition of the cluster C can be explained by the fact that the Brown Swiss and Kostroma breeds have long been actively employed in the historical breeding processes related to the Kyrgyz cattle, which in turn, we believe, have been used in the formation of the Altai cattle. It is known that the Altai cattle continue to exist thanks to the Altai ethnic group called ‘Telengits’, who are the closest relatives of the Kyrgyz people and share a common cultural context and language with them. It is very likely that, to keep up the Altai cattle population, Kyrgyz native cattle were used. However, a more detailed analysis is required.

The other cow samples, Yaroslavl and Red Gorbatov, appear as separate independent breeds: even though the samples cluster among themselves, they do so with little bootstrap support (45.9%). From among the 18 cattle breeds being studied, the greatest genetic distance from the common ancestor (root) was observed for the Yakut breed. This fact, combined with low heterozygosity (Table 3), is explained by the geographical isolation of the breeding territory [27,39]. The Ukrainian Grey, Khogorogo, Yaroslavl, Kostroma, and Altai cattle breeds were slightly less different from their common ancestor. This fact indicates that each of these breeds has a unique gene pool. Overall, the topology of the dendrogram is consistent with our data on the breeds’ origin and does not contradict the results of the Bayesian clustering analysis.

## 4. Discussion

In recent years, interest in the genetics of indigenous livestock breeds with low population sizes has grown. There is no doubt that native cattle breeds are important genetic resources, since they possess unique gene pools that arise from long-term adaptation to the local ecological, social, and economic conditions [27,66,67]. Forming in parallel with the development of the agricultural society during the thousands of years of the human history, local cattle have not been subjected to intensive directional selection for production traits. Therefore, the level of genetic diversity in them may be higher than in commercial breeds. Unfortunately, some native breeds are in danger of extinction and their genetic diversity is compromised due to the production growth, and rapidly changing social and ecological conditions. Conservation of native cattle breeds is necessary to preserve these diverse gene pools. It should be noted that protective measures are also required for some commercial ones, in particular, for Yaroslavl, Kholmogory, and Red Gorbatov cattle, to maintain their breeding value. For these proposes, sufficient knowledge of the genetic population structure, diversity, and evolutionary origin of breeds is needed.

Using the microsatellite data, we studied 18 *Bos taurus* breeds from Eastern Europe and Central East Asia, to assess the genetic diversity and phylogenetic relationships between them. The cattle samples studied belong to various eco-geographic regions and have different climatic and farming conditions and production purposes. Eleven of them are native cattle breeds. We selected breeds based on the likely historical contribution of local cattle specimens to their modern genomes. Along with the popular numerous Russian breeds (e.g., Kholmogory and Yaroslavl), we included highly specialized breeds that show extensive adaptation to specific conditions (e.g., Yakut) or are nearly extinct (e.g., Buryat and Altai). Thus, our study considers the complete set of cattle breeds from Eastern Europe and Central East Asia, in which all the local breeds of Asian origin, namely Altai, Kalmyk, Kyrgyz, Yakut, Khogorogo, Gobi, and Buryat, belong to the Turano-Mongolian type [40]. The main originality of this work is the use of rare and ancient native breeds, which are either unstudied or poorly studied: the Altai, Ukrainian Grey, Tagil, and Buryat cattle breeds. The history of their formation was described above.

We identified breeds with high levels of heterozygosity (>0.77) and allelic richness (>6.6) simultaneously. Such hidden resources of genetic diversity were detected in four the ancient native breeds, namely the Kalmyk, Tagil, Kyrgyz native, and Buryat breeds. We found private alleles at the Ukrainian Grey and all the local breeds of Asian origin, except for the Altai breed, probably due to its small sample size (n = 21). The percent of private alleles per sample, adjusted for sample size, varies from 9.7 to 19.9% of their total number. The presence of private alleles at the native cattle breeds may point to their unique gene pools, and the private alleles may serve as an effective tool for the genetic identification of breeds of the Turano-Mongolian type.

In terms of pairwise Fst values, the greatest genetic dissimilarities revealed in the Yakut and Ukrainian Grey cattle breeds having unique gene pools [27] with others are consistent with the results of phylogenetic analysis, which show the greatest phylogenetic distances from a common ancestor to these cattle breeds. At the same time, in terms of pairwise Ast values, the Yaroslavl and Gobi breeds demonstrated greatest allelic differentiation between themselves, as well as with all other studied breeds. Moreover, average allelic diversity within samples turned out to be higher than that between samples, which suggests positive selection leading to changes in allele frequencies.

Using clustering approaches, we confirmed that the Kostroma, Brown Swiss, and Alatau breeds have a close genetic relationship corresponding to their historical formation [27,63]. Moreover, we found that the Altai cattle is genetically close to the Kyrgyz native cattle and to three breeds developed on its basis, namely the Kyrgyz beef-type, Aulie-Ata, and Alatau ones, the relationship of which was confirmed by the analysis of pairwise Ast. This finding does not contradict our knowledge about the historical formation of Altai cattle. It is known that the Altai breed, which is officially classified as ‘extinct’, continues to exist thanks to the Telengits, who are the closest relatives of the Kyrgyz people and share a similar language and culture, in particular, the culture of breeding cattle, with them. The Kyrgyz cattle were involved to some extent in the process of conserving the Altai cattle and, as a result, the Altai and Kyrgyz cattle have a close genetic kinship. It is noteworthy that the Altai and Kyrgyz breeds, belonging to the Turano-Mongolian type, show a close genetic relationship with the Brown Swiss and Kostroma breeds, but are visibly different from other breeds of the Turano-Mongolian type (the Yakut, Buryat, Kalmyk, Gobi, and Khogorogo breeds). This result can be explained by the fact that extensive crossbreeding with the Brown Swiss and Kostroma breeds has been used to develop the Kyrgyz breeds, which, in turn, have been exploited for breeding the Altai cattle. The Ukrainian Grey breed is the core of a cluster of other East European breeds, in particular the Kholmogory, Holstein, and Tagil cows, which probably indicates a significant influence of the ancestral gene pool of European breeds on the formation of this one. The Yakut and Buryat cattle, together with the Gobi and Khogorogo breeds, as well as with the Kalmyk one, form a separate cluster, which indicates the shared origin of these breeds.

By using the Bayesian approach for clustering analysis, we found a mixed cluster comprising breeds of both European and Asian origins. Perhaps the reason for such clustering is that to incorporate strong and extensive adaptability and to preserve the genetic resources of the Asian breeds such as the Altai, Kalmyk, and Kyrgyz cattle samples, the European breeds (specifically, Brown Swiss and Kostroma) have long been actively employed in historical breeding processes. By using the same approach, we observed that only the Yakut, Ukrainian Grey, Holstein, and Yaroslavl breeds can be considered purebred, since proportion of membership of each breed in a cluster (of 18 clusters) >80%. For other samples, we detected a mixed structure explained by interbreed crossings.

## 5. Conclusions

The estimated genetic diversity of the studied cattle breeds closely reflects the breeding process state on farms. Since we focused on the analysis of poorly explored native breeds, our findings can provide additional important information about the genetic structure and differentiation of cattle. The phylogenetic relationships identified among the cattle breeds, in general, correspond to the historical data on the origin and the results obtained in other researches [27,39]. This study gives new knowledge on the genetic diversity of the European and Asian cattle (*Bos taurus*) breeds, which may be useful in developing future breeding and preservation programs for this species. Nonetheless, further studies using high-density SNP array or whole-genome sequence data are needed to confirm the findings and further explore the gene pool of some of the rare breeds.

## Figures and Tables

**Table 1:** Information on cattle samples.

Breed (Code)	Breeding Purpose	Category	n	Location of Sample	Latitude, Longitude
*European origin*					
Brown Swiss (BrSwis)	Dual	IT *	50	Kostroma region, Kostroma district (Russia)	57.77, 40.93
Holstein (Holst)	Milk	IT	176	Moscow region (Russia)	55.4, 37.27
Kostroma (Kostr)	Dual	RT **	20	Kostroma Region, Kostroma district, (Russia)	57.77, 40.93
Kholmogory (Kholm)	Milk	RT	50	Komi republic, Inta (Russia)	66.03, 60.17
Yaroslavl (Yaros)	Milk	RT	50	Yaroslavl region, Yaroslavl district (Russia)	57.73, 39.83
Tagil (Tagil)	Milk	Native	49	Perm region, Oktyabrsky District (Russia)	56.51, 57.2
Red Gorbatov (RedGor)	Milk	Native	50	Nizhny Novgorod region, Pavlovsky district (Russia)	56.03, 43.16
Ukrainian Grey (Grey)	Working	Native	44	Altai republic, Shebalinsky district (Russia)	51.34, 85.41
*Asian origin*					
Aulie-Ata (AulieAt)	Milk	RT	42	Talas region, Talas District, (Kyrgyzstan)	42.76, 71.41
Alatau (Alatau)	Dual	RT	49	Chui region, Zhayilsky district (Kyrgyzstan)	42.81, 71.41
Kyrgyz Beef-type (KyrgBT)	Meat	Native	48	Chui region, Panfilovsky district (Kyrgyzstan)	42.82, 73.67
Kyrgyz native (KyrgNat)	Dual	Native	49	Naryn region, At-Bashinsky District (Kyrgyzstan)	41.24, 76.13
Yakut (Yakut)	Dual	Native	30	Yakutia republic (Russia)	67.63, 130.86
Altai (Altai)	Dual	Native	21	Altai, Ulagan district, Yazula, (Russia)	50.63, 88.77
Kalmyk (Kalm)	Meat	Native	54	Kalmykia republic, Yustinsky district (Russia)	47.11, 45.97
Khogorogo (Khogor)	Dual	Native	50	Khuvsgul aimag (Mongolia)	46.00, 105.00
Gobi (Gobi)	Milk	Native	50	South Gobi aimag (Mongolia)	43.34, 104.25
Buryat (Buryat)	Dual	Native	24	Khuvsgul aimag (Mongolia)	46.00, 105.00
			10	Inner Mongolia (China)	41.13, 116.38
			252	Buryatia Republic, Dzhidinsky District (Russia)	50.65, 105.22

Notation: *n*: sample size; * International transboundary; ** Regional transboundary.

**Table 2:** Population parameters for the cattle samples studied.

Breed	N	A	%	Ar	Ho	He	Fis	HWE
*European origin*								
Brown Swiss	49.43	79	42.27	5.29	0.77	0.7	−1.00 × 10^−1^	0 × 10^0^
Holstein	176	98	52.57	5.53	0.72	0.71	−1.41 × 10^−2^	8.07 × 10^−1^
Kostroma	20	86	45.55	5.43	0.71	0.68	−4.41 × 10^−2^	6.16 × 10^−1^
Kholmogory	49.86	95	50.9	5.79	0.73	0.73	0 × 10^0^	9.80 × 10^−2^
Yaroslavl	50	89	48.36	5.56	0.72	0.7	−2.86 × 10^−2^	6.59 × 10^−1^
Tagil	48.21	113	60.19	6.69	0.77	0.76	−1.32 × 10^−2^	7.04 × 10^−2^
Red Gorbatov	50	109	58.02	6.21	0.78	0.73	−6.85 × 10^−2^	9.99 × 10^−1^
*Asian origin*								
Aulie−Ata	40.71	129	69.11	7.21	0.75	0.76	1.32 × 10^−2^	5.70 × 10^−3^
Alatau	49	132	70.13	7.52	0.76	0.76	0 × 10^0^	4.58 × 10^−1^
Kyrgyz Beef−type	48	115	61.96	6.77	0.75	0.74	−1.35 × 10^−2^	1.40 × 10^−1^
Kyrgyz native	48.93	128	66.93	7.23	0.77	0.76	−1.32 × 10^−2^	9.75 × 10^−1^
Yakut	30	66	35.9	4.29	0.61	0.58	−5.17 × 10^−2^	9.53 × 10^−1^
Altai	21	87	46.71	5.57	0.71	0.7	−1.43 × 10^−2^	6.60 × 10^−1^
Kalmyk	54	131	69.8	7.51	0.78	0.78	0 × 10^0^	2.00 × 10^−4^
Khogorogo	50	98	52.38	5.8	0.69	0.69	0 × 10^0^	4.51 × 10^−2^
Gobi	50	99	52.16	6.12	0.75	0.75	0 × 10^0^	6.50 × 10^−1^
Buryat	285.64	155	81.51	7.36	0.77	0.78	1.28 × 10^−2^	0 × 10^0^

Notation: N: the average number of individuals genotyped by the locus; A: the number of alleles per sample; %: the average percentage of the total number of alleles observed in the locus; Ar: the mean allelic richness across loci; Ho and He: the observed and expected heterozygosity, correspondently; HWE: the *p*-value for the chi-square testing the Hardy–Weinberg equilibrium; Fis: fixation index.

**Table 3:** Private breed-specific alleles by locus and sample.

Breed	% *	Locus	Allele	AF
Grey (1) **	11.1	BM1824	176	0.011
Buryat (9)		CSSM66	177	0.002
		ILSTS006	276	0.019
		TGLA227	69	0.009
		TGLA227	71	0.005
	15.5	TGLA122	137	0.002
		SPS115	238	0.002
		ETH225	160	0.031
		TGLA53	190	0.007
		BM1824	192	0.011
Gobi (1)	9.7	TGLA227	85	0.020
Kalm (2)	18	TGLA126	129	0.028
		CSRM60	86	0.009
Khogor (1)	9.7	CSRM60	88	0.040
KyrgNat (2)	19.9	CSSM66	207	0.010
		INRA023	194	0.010

Notation: * percent of private alleles per sample, adjusted for sample size; ** number of private alleles per population sample.

**Table 4:** Summary of Wright’s F-statistics for each locus.

Locus	Fst(se)	Fis(se)	Fit(se)
ILSTS006	0.0955(0.0132)	−0.0022 (0.0312)	0.0935 (0.0306)
SPS115	0.0896(0.0140)	−0.0019 (0.0336)	0.0879 (0.0333)
BM1824	0.0837(0.0111)	0.0041 (0.0346)	0.0875 (0.0332)
TGLA122	0.0821(0.0107)	−0.0137 (0.0257)	0.0695 (0.0255)
ETH3	0.0793(0.0108)	−0.0040 (0.0326)	0.0756 (0.0318)
ETH225	0.0781(0.0099)	−0.0169 (0.0312)	0.0625 (0.0297)
BM2113	0.0762(0.0099)	−0.0050 (0.0288)	0.0716 (0.0278)
TGLA126	0.0759 (0.0123)	−0.0086 (0.0374)	0.0680 (0.0364)
CSSM66	0.0740 (0.0096)	−0.0185 (0.0258)	0.0568 (0.0252)
TGLA53	0.0715 (0.0079)	0.0036 (0.0222)	0.0749 (0.0220)
INRA023	0.0694 (0.0105)	−0.0047 (0.0285)	0.0651 (0.0282)
CSRM60	0.0659 (0.0094)	−0.0349(0.0289)	0.0333 (0.0287)
ETH10	0.0615 (0.0109)	−0.0215(0.0320)	0.0413 (0.0317)
TGLA227	0.0608 (0.0077)	0.0059(0.0236)	0.0663 (0.0234)

## Appendix A

**Table A1:** Description of microsatellite markers.

Locus and Source Reference	Position on Chromosome	Repeating Sequences	Sequences Forward (F) and Inverse (R) Primers	Length of Amplicons (bp)
BM1824 [68]	D1S34	(GT)n	F: GAGCAAGGTGTTTTTCCAATC R: CATTCTCCAACTGCTTCCTTG	176–188
BM2113 [69]	D2S26	(CA)n	F: GCTGCCTTCTACCAAATACCC R: CTTCCTGAGAGAAGCAACACC	124–146
CSRM60 [70]	D10S5	(AC)n	F: AAGATGTGATCCAAGAGAGAGGCA R: AGGACCAGATCGTGAAAGGCATAG	91–117
CSSM66 [68]	D14S31	(AC)n	F: AATTTAATGCACTGAGGAGCTTGG R: ACACAAATCCTTTCTGCCAGCTGA	177–203
ETH3 [71]	D19S2	(GT)nAC(GT)_6_	F: GAACCTGCCTCTCCTGCATTGG R: ACTCTGCCTGTGGCCAAGTAGG	100–128
ETH10 [71]	D5S3	(AC)n	F: GTTCAGGACTGGCCCTGCTAACA R: CCTCCAGCCCACTTTCTCTTCTC	206–222
ETH225 [72]	D9S2	(TG)_4_CG(TG)(CA)n	F: GATCACCTTGCCACTATTTCCT R: ACATGACAGCCAGCTGCTACT	139–157
ILSTS006 [73]	D7S8	(GT)n	F: TGTCTGTATTTCTGCTGTGG R: ACACGGAAGCGATCTAAACG	279–297
INRA023 [74]	D3S10	(AC)n	F: GAGTAGAGCTACAAGATAAACTTC R: TAACTACAGGGTGTTAGATGAACTC	201–225
SPS115 [75]	D15	(CA)nTA(CA)_6_	F: AAAGTGACACAACAGCTTCACCAG R: AACCGAGTGTCCTAGTTTGGCTGTG	247–261
TGLA53 [76]	D16S3	(TG)_6_CG(TG)_4_(TA)n	F: GCTTTCAGAAATAGTTTGCATTCA R: ATCTTCACATGATATTACAGCAGA	151–187
TGLA122 [76]	D21S6	(AC)n(AT)n	F: AATCACATGGCAAATAAGTACATAC R: CCCTCCTCCAGGTAAATCAGC	136–182
TGLA126 [76]	D20S1	(TG)n	F: CTAATTTAGAATGAGAGAGGCTTCT R: TTGGTCCTCTATTCTCTGAATATTCC	111–127
TGLA227 [76]	D18S1	(TG)n	F: GGAATTCCAAATCTGTTAATTTGCT R: ACAGACAGAAACTCAATGAAAGCA	76–104

**Table A2:** The *p*-values for the Hardy–Weinberg equilibrium test for each combination of sample and locus.

Breed	ETH3	CSSM66	INRA023	ILSTS006	TGLA227	TGLA126	TGLA122	SPS115	ETH225	TGLA53	CSRM60	BM2113	BM1824	ETH10
**Holst**	0.142	0.743	0.546	0.617	0.721	0.429	0.479	0.433	0.394	0.134	0.986	0.77	0.225	0.849
**Tagil**	0.354	0.411	0.969	0.411	0.493	0.559	0.595	0.293	0.133	0.28	0.725	0.465	0.342	0.298
**Kholm**	0.325	0.348	0.141	0.844	0.058	0.818	0.181	0.277	0.106	0.114	0.794	0.646	0.673	0.212
**RedGor**	0.6	0.267	0.241	0.668	0.5	0.005	0.282	0.614	0.767	0.776	0.502	0.832	0.282	0.45
**BrSwis**	0.102	0	0.314	0.004	0	0.025	0.001	0.623	0.001	0	0.006	0	0.07	0.163
**Grey**	0.321	0.331	0.764	0.341	0.737	0.889	0.844	0.184	0.295	0.917	0.457	0.666	0.14	0.57
**Yaros**	0.212	0.224	0.986	0.135	0.322	0.865	0.867	0.352	0.186	0.182	0.155	0.855	0.559	0.294
**Kostr**	0.497	0.562	0.025	0.625	0.092	1	0.397	0.701	0.169	0.267	0.233	0.823	0.1	0.364
**Altai**	0.008	0.301	0.012	0.257	0.409	0.694	0.914	0.029	0.538	0.282	0.911	0.84	0.953	0.494
**Kalm**	0.464	0.084	0.048	0.491	0.473	0.239	0.555	0.103	0.184	0.122	0.565	0.165	0.098	0.861
**AulieAt**	0.799	0.09	0.182	0.071	0.405	0.391	0.74	0.333	0.321	0.844	0.808	0.054	0.694	0.68
**Alatau**	0.844	0.207	0.334	0.975	0.647	0.652	0.015	0.094	0.247	0.015	0.626	0.5	0.594	0.527
**KyrgBT**	0.089	0.909	0.319	0.837	0.504	0.007	0.121	0.027	0.337	0.483	0.22	0.712	0.888	0.32
**KyrgNat**	0.094	0.465	0.898	0.218	0.53	0.541	0.927	0.337	0.418	0.372	0.304	0.731	0.833	0.927
**Yakut**	0.085	0.785	0.359	0.918	0.018	0.981	0.005	1	0.619	0.755	0.619	0.9	0.559	0.91
**Khogor**	0.345	0.912	0.269	0.005	0.095	0.115	0.037	0.155	0.605	0.465	0.186	0.697	0.456	0.348
**Gobi**	0.301	0.392	0.25	0.067	0.156	0.579	0.457	0.146	0.248	0.595	0.84	0.388	0.14	0.315
**Buryat**	0.761	0.133	0.552	0	0.898	0.321	0.017	0.819	0.034	0.048	0.322	0.029	0.969	0.578

**Table A3:** The polymorphism information content values for each combination of sample and locus.

Breed	ETH3	CSSM66	INRA023	ILSTS006	TGLA227	TGLA126	TGLA122	SPS115	ETH225	TGLA53	CSRM60	BM2113	BM1824	ETH10
**Holst**	0.628	0.731	0.713	0.534	0.789	0.542	0.826	0.545	0.644	0.805	0.646	0.662	0.633	0.660
**Tagil**	0.646	0.803	0.668	0.726	0.850	0.564	0.856	0.603	0.723	0.851	0.632	0.783	0.695	0.721
**Kholm**	0.710	0.736	0.788	0.727	0.774	0.705	0.533	0.700	0.550	0.869	0.633	0.728	0.637	0.592
**RedGor**	0.704	0.788	0.725	0.737	0.787	0.558	0.757	0.399	0.771	0.804	0.637	0.759	0.745	0.601
**BrSwis**	0.590	0.771	0.492	0.723	0.830	0.462	0.552	0.471	0.785	0.862	0.638	0.682	0.636	0.676
**Grey**	0.621	0.627	0.776	0.672	0.519	0.544	0.626	0.481	0.450	0.581	0.696	0.769	0.571	0.505
**Yaros**	0.671	0.784	0.693	0.683	0.737	0.579	0.746	0.260	0.733	0.661	0.763	0.705	0.608	0.617
**Kostr**	0.445	0.822	0.629	0.666	0.804	0.386	0.655	0.541	0.649	0.784	0.571	0.745	0.559	0.571
**Altai**	0.462	0.642	0.725	0.528	0.784	0.467	0.666	0.637	0.680	0.881	0.726	0.723	0.677	0.684
**Kalm**	0.750	0.806	0.832	0.736	0.806	0.692	0.746	0.630	0.780	0.871	0.769	0.843	0.659	0.614
**AulieAt**	0.710	0.851	0.705	0.734	0.852	0.550	0.635	0.645	0.719	0.833	0.734	0.789	0.687	0.771
**Alatau**	0.720	0.820	0.705	0.734	0.862	0.589	0.803	0.555	0.738	0.874	0.638	0.806	0.704	0.718
**KyrgBT**	0.575	0.838	0.755	0.722	0.844	0.586	0.728	0.572	0.683	0.806	0.734	0.774	0.676	0.604
**KyrgNat**	0.662	0.800	0.690	0.726	0.852	0.568	0.827	0.653	0.743	0.882	0.635	0.822	0.700	0.653
**Yakut**	0.563	0.629	0.371	0.675	0.688	0.667	0.455	0.303	0.651	0.538	0.482	0.627	0.240	0.540
**Khogor**	0.597	0.782	0.632	0.559	0.791	0.536	0.812	0.581	0.544	0.794	0.812	0.668	0.411	0.550
**Gobi**	0.677	0.698	0.717	0.729	0.858	0.547	0.687	0.619	0.716	0.838	0.808	0.762	0.669	0.600
**Buryat**	0.728	0.768	0.810	0.709	0.856	0.632	0.804	0.691	0.706	0.911	0.727	0.765	0.660	0.658
**Overall**	**0.740**	**0.837**	**0.790**	**0.763**	**0.870**	**0.643**	**0.822**	**0.647**	**0.759**	**0.903**	**0.756**	**0.817**	**0.706**	**0.692**

**Table A4:** Nei’s Pairwise Fst values among samples.

Breed	Holst	Tagil	Kholm	RedGor	BrSwis	Grey	Yaros	Kostr	Altai	Kalm	AulieAt	Alatau	KyrgBT	KyrgNat	Yakut	Khogor	Gobi
**Tagil**	0.0163																
**Kholm**	0.0333	0.0325															
**RedGor**	0.033	0.0268	0.0417														
**BrSwis**	0.0329	0.0321	0.0558	0.0431													
**Grey**	0.047	0.0628	0.0665	0.0779	0.0876												
**Yaros**	0.0498	0.0407	0.0583	0.0468	0.0587	0.0922											
**Kostr**	0.0229	0.0367	0.0567	0.0416	0.0319	0.087	0.0654										
**Altai**	0.0268	0.0359	0.0522	0.0372	0.0426	0.0836	0.0588	0.0489									
**Kalm**	0.0268	0.0155	0.0322	0.0291	0.0306	0.0652	0.0341	0.0351	0.0323								
**AulieAt**	0.0208	0.0182	0.0312	0.0308	0.0311	0.0687	0.0476	0.0275	0.0348	0.0204							
**Alatau**	0.0235	0.0193	0.0398	0.0283	0.0276	0.071	0.0508	0.0244	0.025	0.0163	0.0198						
**KyrgBT**	0.0301	0.0281	0.0472	0.0311	0.0304	0.0658	0.0587	0.0231	0.0298	0.028	0.0272	0.0136 ***					
**KyrgNat**	0.0267	0.0198	0.0402	0.031	0.0291	0.0696	0.0535	0.0271	0.0291	0.0156	0.0204	0.0049*	0.0147				
**Yakut**	0.0634	0.0796	0.0953	0.1	0.1161	0.1459	0.1127	0.1527	0.1275	0.0761	0.0913	0.0912	0.1102	0.0803			
**Khogor**	0.0512	0.0409	0.0645	0.059	0.0762	0.1074	0.0667	0.0815	0.0642	0.0352	0.0523	0.0503	0.0695	0.0487	0.0763		
**Gobi**	0.0338	0.0238	0.05	0.0342	0.0525	0.066	0.0531	0.0494	0.0369	0.0209	0.0328	0.0266	0.0391	0.0251	0.0675	0.0299	
**Buryat**	0.0385	0.0087 *	0.0173	0.0188	0.0218	0.0317	0.0242	0.0127 **	0.0118 **	0.0073 *	0.0108 **	0.0097 *	0.0165	0.0094 *	0.0231	0.0136 **	**0.0089 ***

Notation: the lower triangle shows the pairwise Fst values. The pairwise Fst values marked by *, ** or *** show non-significant differences with *p*-values greater than 0.05, 0.01, or 0.005, respectively.

**Table A5:** Allelic distances among samples.

	Holst	Tagil	Kholm	RedGor	BrSwis	Grey	Yaros	Kostr	Altai	Kalm	AulieAt	Alatau	KyrgBT	KyrgNat	Yakut	Khogor	Gobi
**Tagil**	18.5																
**Kholm**	19.5	20															
**RedGor**	18.5	21	22														
**BrSwis**	19.5	25	21	21													
**Grey**	29.5 *	30 *	28 *	31 *	25												
**Yaros**	24.5	25	23	22	23	31 **											
**Kostr**	25	24.5	18.5	23.5	18.5	26.5 *	22.5										
**Altai**	24.5	29 *	22	23	23	27 *	23	21.5									
**Kalm**	29.5 *	24	24	21	31 **	35 **	29 *	28.5 *	27 *								
**AulieAt**	23.5	24	29 *	22	32 **	33 **	29 *	31.5 **	30 **	25 *							
**Alatau**	26	25.5	27.5 *	23.5	29.5*	36.5**	27.5*	27 *	28.5 **	16.5	21.5						
**KyrgBT**	24.5	19	17	22	26 *	33 **	25 *	23.5	26 *	23	23	19.5					
**KyrgNat**	28	23.5	25.5	19.5	27.5 *	36.5 **	29.5 *	26 *	27.5 *	19.5	23.5	14	19.5				
**Yakut**	30 *	29.5 *	23.5	30.5 *	25.5	30.5 **	24.5	27 **	26.5 *	37.5 **	39.5 **	39 **	32.5 **	39 **			
**Khogor**	28	26.5	27.5 *	26.5	25.5	31.5 *	26.5 *	28 *	28.5 **	30.5 **	27.5 **	28 **	26.5 **	28 **	29 **		
**Gobi**	26.5	24	22	20	22	30 *	25	20.5	20	23	28 **	22.5	23	22.5	26.5 *	20.5	
**Buryat**	34.5 *	29	35 *	32 *	41 **	45 **	38 **	38.5 **	37 **	25	27 *	24.5	30 **	26.5 *	48.5 **	36.5 **	33 **

Notation: the lower triangle shows allelic distances (Adis). The pairwise Adis values marked by *, ** or *** show non-significant differences with *p*-values greater than 0.05, 0.01, or 0.005, respectively.

**Table A6:** Pairwise indices of allelic differentiation among samples.

	Holst	Tagil	Kholm	RedGor	BrSwis	Grey	Yaros	Kostr	Altai	Kalm	AulieAt	Alatau	KyrgBT	KyrgNat	Yakut	Khogor	Gobi
**Tagil**	0.2259																
**Kholm**	0.1895	0.2357															
**RedGor**	0.2431	0.2536	0.2524														
**BrSwis**	0.2043	0.235	0.218	0.2369													
**Grey**	0.2067	0.2144	0.2264	0.238	0.2058												
**Yaros**	0.2984	0.2935	0.2908	0.2892	0.2976	0.2856											
**Kostr**	0.262	0.2639	0.2349	0.2523	0.2555	0.2679	0.315										
**Altai**	0.1809	0.2156	0.2069	0.2467	0.1926	0.205	0.2992	0.27									
**Kalm**	0.2438	0.2828	0.2514	0.2823	0.2452	0.2296	0.3215	0.2706	0.248								
**AulieAt**	0.234	0.2288	0.2329	0.2441	0.228	0.244	0.2853	0.239	0.2264	0.2806							
**Alatau**	0.2162	0.23	0.2392	0.2202	0.2379	0.2228	0.29	0.2848	0.2192	0.2882	0.2198						
**KyrgBT**	0.1765	0.2322	0.2001	0.2311	0.2048	0.2171	0.284	0.2501	0.1978	0.2512	0.1984	0.1989					
**KyrgNat**	0.1602	0.2276	0.2023	0.2363	0.2017	0.2029	0.3028	0.2717	0.1854	0.2465	0.2269	0.2129	0.1799				
**Yakut**	0.2096	0.2333	0.2048	0.2288	0.2278	0.2166	0.2956	0.2276	0.2075	0.2601	0.2198	0.2367	0.2053	0.2066			
**Khogor**	0.2155	0.2486	0.1971	0.2333	0.2017	0.2199	0.2847	0.2098	0.2058	0.2447	0.1971	0.2294	0.1913	0.2091	0.195		
**Gobi**	0.3141	0.3047	0.3102	0.3118	0.2991	0.2775	0.3447	0.3185	0.3081	0.3132	0.2747	0.3092	0.2982	0.3095	0.297	0.2768	
**Buryat**	0.2417	0.2396	0.2509	0.2592	0.2494	0.245	0.3089	0.2684	0.238	0.2763	0.2459	0.2413	0.2271	0.2584	0.233	0.2408	0.2895
